# A Prospective Study to Evaluate the Impact of Golimumab Therapy on Work Productivity and Activity, and Quality of Life in Patients With Rheumatoid Arthritis, Psoriasis Arthritis and Axial Spondyloarthritis in a Real Life Setting in AUSTRIA. The GO-ACTIVE Study

**DOI:** 10.3389/fmed.2022.881943

**Published:** 2022-06-02

**Authors:** Christian Dejaco, Thomas Mueller, Omid Zamani, Ulrike Kurtz, Stefan Egger, Johannes Resch-Passini, Anna Totzauer, Babak Yazdani-Biuki, Thomas Schwingenschloegl, Peter Peichl, Angelika Kraus, Gerhard W. Naerr

**Affiliations:** ^1^Department of Rheumatology, Medical University of Graz, Graz, Austria; ^2^Department of Rheumatology, Hospital of Brunico (SABES-ASDAA), Brunico, Italy; ^3^Private Office, Graz, Austria; ^4^Rheuma Zentrum Favoriten, Vienna, Austria; ^5^Rheuma Praxis Dr. Kurtz, Gleisdorf, Austria; ^6^Ordination Gesundheitsquadrat, Vienna, Austria; ^7^Rheuma-Zentrum Wien-Oberlaa GmbH, Vienna, Austria; ^8^Private Office Dr. Anna Totzauer, Gmünd, Austria; ^9^Dr. Schrenk & Dr. Yazdani-Biuki OG - Gruppenpraxis, Fürstenfeld, Austria; ^10^Private Office Dr. Thomas Schwingenschloegl, Wiener Neudorf, Austria; ^11^Private Office Prim. Univ. Doz. Dr. Peter Peichl, Vienna, Austria; ^12^Private Office Dr. Angelika Kraus, Melk, Austria; ^13^MSD Ges.m.b.H., The Icon Vienna, Vienna, Austria

**Keywords:** socio economic, health economic, biologicals, rheumatoid arthritis, psoriatic arthritis, ankylosing spondylitis, TNF inhibitor, golimumab

## Abstract

**Objective:**

To analyze real-world evidence on work productivity and daily activity impairment (WPAI) and health-related quality of life (HRQoL) in rheumatoid arthritis (RA), psoriatic arthritis (PsA), and axial spondyloarthritis (axSpA) patients treated with golimumab in Austria.

**Methods:**

This was a prospective, non-interventional, multi-center study conducted in RA, PsA and axSpA patients initiating golimumab between April 2016 and May 2020 in 40 centers in Austria. WPAI, HRQoL (RAQoL, ankylosing spondylitis (AS)QoL and PsAQoL) questionnaires and disease activity (Clinical Disease Activity Index, CDAI, in RA and PsA; Bath Ankylosing Spondylitis Disease Activity Index, BASDAI, in axSpA) were assessed at baseline and months 3, 6, 12, 18, and 24. Association between WPAI and disease activity was tested using linear regression.

**Results:**

We enrolled 233 patients (RA, *n* = 95; axSpA, *n* = 69; PsA, *n* = 69), 110 patients were followed up to month 24. Mean age was 50.2 ± 14.2 years; 64% were female. Disease activity decreased from baseline to month 24 (RA: CDAI −24.3 ± 13.5; axSpA: BASDAI −4.4 ± 2.1, and PsA: CDAI −21.7 ± 8.5, *p* < 0.0001, each). Total work productivity impairment (TWPI), activity impairment and presenteeism subscores continuously decreased throughout month 24 in all indications: RA (−58.3 ± 23%, −62.6 ± 23.8% and −61.7 ± 23.3%, respectively as compared to baseline; *p* < 0.0001, each), axSpA (−34.4 ± 38.3%, *p* = 0.0117; −60.9 ± 25.9%, and −43.8 ± 26.6%, respectively, *p* ≤ 0.0001 both) and PsA (−35.8 ± 43.7%, *p* = 0.0186; −52.3 ± 25.4%, *p* < 0.0001; and −43.3 ± 33.5%, *p* = 0.0007, respectively). Absenteeism scores decreased only in RA patients (−9.2 ± 24.9%, *p* = 0.0234). HRQoL improved between baseline and month 24 (RAQoL: −12.6 ± 7.5; ASQoL: −8.0 ± 4.3; PsAQoL; −8.3 ± 6.4, *p* < 0.0001, each). TWPI, presenteeism and activity impairment strongly associated with disease activity throughout the study.

**Conclusions:**

This real-world study confirms the benefit of golimumab on work productivity/daily activity impairment in Austrian RA, PsA, and axSpA patients.

## Introduction

Rheumatoid arthritis (RA), axial spondyloarthritis (axSpA) and psoriatic arthritis (PsA) are chronic inflammatory rheumatic diseases characterized by pain, swelling, fatigue and stiffness as well as structural changes of bone and cartilage (1). Patients with these diseases often suffer from physical and psychological restrictions leading to a reduced quality of life (QoL) (2–4). Furthermore, many patients with RA, axSpA and PsA have a significant lower ability to work (5–7). For example, it was shown that within 9 years from the onset of symptoms, 37% of employed RA patients became work-disabled due to disease (8). This is not only a substantial financial burden for patients but causes also high indirect costs to the society.

Tumor necrosis factor α (TNFα) is an inflammatory cytokine mediating the destruction and pathological remodeling within the joints. TNFα blockade has been shown to preserve joint structure and to maintain its function in several forms of chronic arthritis (9–11). Golimumab is an anti-TNFα antibody approved for the use in adults with RA, axSpA and PsA (12). The efficacy and safety of golimumab was demonstrated in several large scale randomized controlled clinical trials (13–15) and non-interventional studies (16–18). Furthermore, currently available real-world evidence showed that golimumab improves QoL and work productivity (17, 19).

Nevertheless, factors determining the work productivity in golimumab-treated patients remain underexplored. In particular, it is currently unknown to what extent improvement in disease activity translates into a better productivity at work. To address this question, this study was designed for the collection and analysis of real- world evidence on work productivity and daily activity impairment, disease activity and additionally health-related quality of life (HRQoL) in Austrian RA, axSpA and PsA patients treated with golimumab over a 2 years period.

## Methods

### Patients and Setting

This was a prospective, non-interventional, multi-center study conducted between April 2016 and May 2020 in 40 centers (hospital based or private practice) in different regions of Austria. Consecutive adult patients with a diagnosis of RA, axSpA or PsA who started treatment with golimumab were included. There was no specific treatment protocol; biologic naïve or biologic-experienced patients could equally be included. Golimumab was administered at a dose and frequency specified in the summary of product characteristics; use of other treatments including conventional synthetic disease modifying anti-rheumatic drugs (csDMARDs) and glucocorticoids was at the discretion of the investigator. Exclusion criteria were previous treatment with golimumab, switch to golimumab from former biologic treatment because of serious adverse events (SAE), opportunistic infection, or allergic reaction, current participation in an interventional trial, hypersensitivity to golimumab, active tuberculosis or other opportunistic infection and/or active Hepatitis B, or other current severe infections. Patients with moderate or severe heart failure (NYHA class III/IV) were also excluded.

Each patient attended 6 visits over a period of 24 months (baseline, months 3, 6, 12, 18, and 24). Patients were followed for up to 2 years or until they discontinued golimumab. The data has been collected through electronic data capture system.

The primary objective of the study was to evaluate the change of impairments in work and daily activities from baseline to month 3 using the Work Productivity and Activity (WPAI) questionnaire. The primary endpoint was analyzed for the entire study population, secondary analyses investigated the outcome in RA, axSpA and PsA patients separately and in biologics naïve and biologics pre-treated patients. The secondary objective was the evaluation of the change of QoL scores from baseline.

Exploratory objectives included evaluation of changes in WPAI questionnaire and change in disease activity.

The study was approved by the Leading Ethics Committee at the Medical University Graz and the Ethics Committee of the Federal State of Carinthia (approval number: 28–312 ex 15/16). Written informed consent was obtained from each participant.

### Assessments

At each study visit, patients' disease activity was determined by the Clinical Disease Activity Index (CDAI) in RA and PsA patients, as well as by the Bath Ankylosing Spondylitis Disease Activity Index (BASDAI) in axSpA patients. In addition, HRQoL was assessed at each visit using the following questionnaires: RAQoL (20, 21) in RA patients, Ankylosing Spondylitis Quality of Life questionnaire (ASQoL, (22) in axSpA patients and PsAQoL (23) in PsA patients.

Patients filled out the WPAI questionnaire at each study visit. The WPAI questionnaire has been validated for RA and axSpA (24, 25) and has previously been used in drug trials to measure productivity gain in RA, axSpA and PsA patients (17, 25–28). Six questions of WPAI determine the employment status (Q1, yes/no), number of working hours missed due to disease (Q2) or other reasons (Q3), number or hours actually worked (Q4), and the degree to which the disease affected work productivity and activities outside of work (Q5 and Q6, respectively, assessed on 0–10 numeric rating scale, with a higher number indicating a greater impairment, see also Supplementary Material 1). The following subscores resulting from the WPAI questionnaire were calculated as described previously (24): Total work productivity impairment due to health (TWPI) using questions Q2, Q4 and Q5 from the WPAI and the following formula: Q2/(Q2 + Q4) + [(1–(Q2/(Q2 + Q4))) x (Q5/10)]. Absenteeism (work time missed due to health) was calculated by Q2/(Q2+Q4), Presenteeism (Impairment while working due to health) by Q5/10 and activity impairment by Q6/10. The WPAI domains TWPI, absenteeism and presenteeism were analyzed in employed patients whereas activity impairment was analyzed in all patients.

Treatment-emergent adverse event (TEAE) and serious non-TEAE, serious TEAE and drug-related TEAE were collected and coded using the Medical Dictionary for Regulatory Activities (MedDRA).

### Statistical Analysis

The study was powered to detect a 12% change in TWPI after 3 months compared to baseline assuming a 20% drop-out rate (no correlation between baseline and 3- month values, paired *t*-test, 80% power, two-sided α = 0.05). Descriptive statistics were used to summarize the data. For continuous data, we present either the mean and standard deviation (parametric distribution of data) or the median and range (non-parametric distribution); categorical data are summarized using absolute and relative frequencies. We used the Wilcoxon signed-rank test for the primary and the secondary objective analyses and Fisher's exact test for exploratory analyses.

Missing data in WPAI and QoL questionnaires were handled according to the recommendations in manual for the specific score (20, 22, 23, 29). All other missing values were not imputed. Univariate linear regression analysis was used to determine whether disease activity scores (CDAI and BASDAI, independent variables) were associated with higher WPAI sub-scores (dependent variables). A positive (or negative) estimate value describes the increase (or decrease) in WPAI sub-scores per one point increase in CDAI or BASDAI score. Univariate linear regression analysis was also used to determine the impact of age, gender, HRQoL, prior use of biologics and employment status at baseline on WPAI sub-scores. *p*-values below 0.05 were considered statistically significant; *p*-values were not adjusted for multiple testing due to the exploratory nature of the analysis.

We used the SAS version 9.4 software to conduct all statistical analyses.

## Results

### Patient Characteristics at Baseline

**Figure 1:**
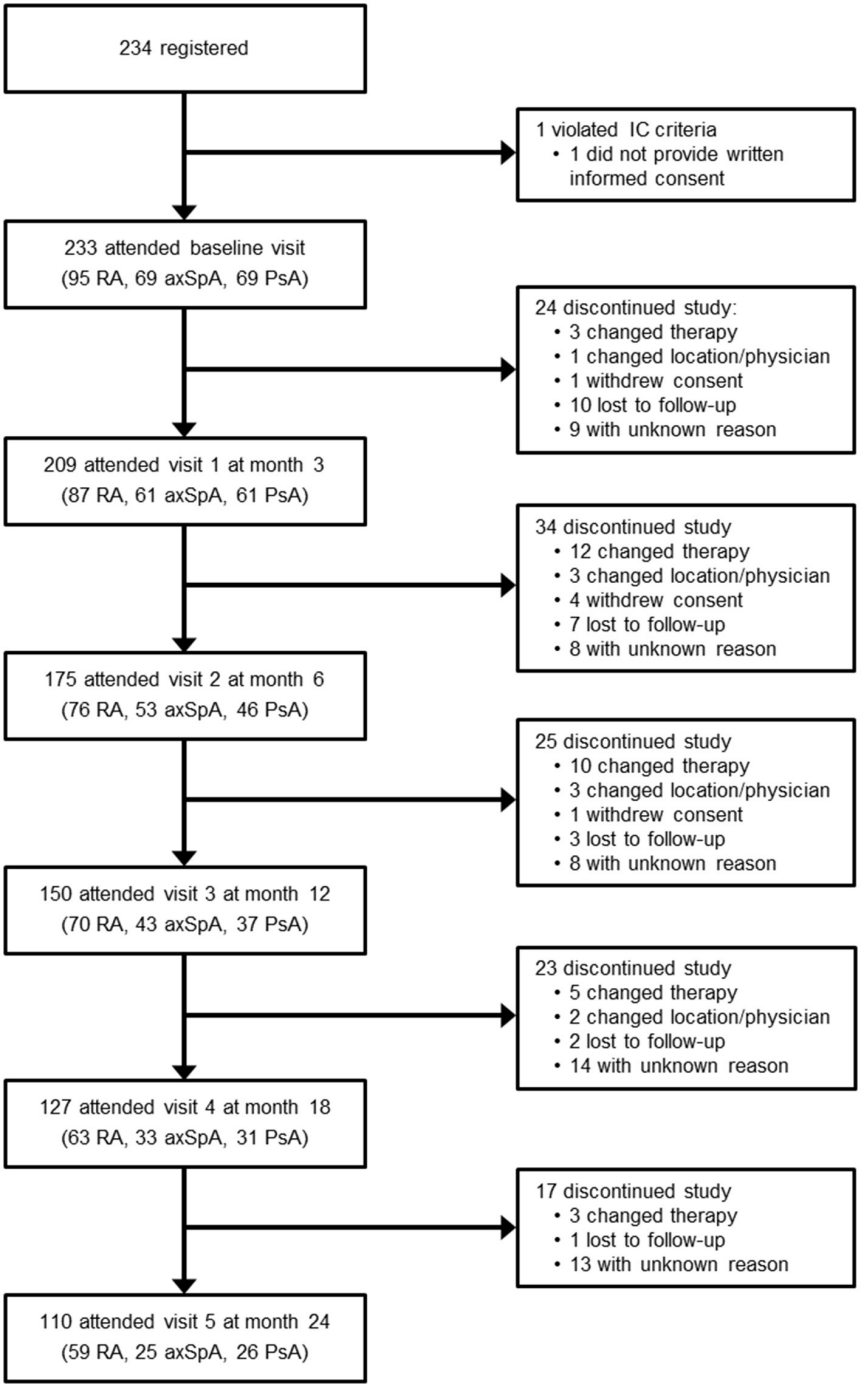
Consort diagram.

Among the 233 patients enrolled, 95 had RA (40.8%), 69 had axSpA (29.6%) and 69 had PsA (29.6%, Table 1). 209 patients (89.7%) attended visit 1 at month 3; 110 patients (47.2%) remained in the study throughout visit 5 at month 24 (Figure 1). 123 patients discontinued the study; the most frequent reason for premature study discontinuation was switch to another treatment (*n* = 33, 26.8%) and lost to follow-up (*n* = 23, 18.7%). 16 out of 233 enrolled patients (6.9%) stopped golimumab therapy.

**Table 1:** Patient characteristics at baseline.

	**RA (*n* = 95)**	**axSpA (*n* = 69)**	**PsA (*n* = 69)**	**Total (*n* = 233)**
**Age [years], mean (SD)**	54.8 (15.3)	42.5 (12.4)	51.6 (10.9)	50.2 (14.2)
**Female**, ***n*** **(%)**	78 (82.1)	30 (43.5)	41 (59.4)	149 (64.0)
**Unemployed**, ***n*** **(%)**	46 (48.4)	16 (23.2)	18 (26.1)	80 (34.3)
**BMI, median (Q1, Q3)**	24.7 (22.2, 28.6)	24.8 (22.9, 29.1)	26.8 (24.5, 30.8)	25.3 (23.1, 29.5)
**Comorbidities**, ***n*** **(%)**				
0	54 (56.8)	45 (65.2)	44 (63.8)	143 (61.4)
1–2	28 (29.5)	20 (29.0)	16 (23.2)	64 (27.5)
3–5	12 (12.6)	3 (4.4)	5 (7.3)	20 (8.6)
≥6	1 (1.1)	1 (1.5)	4 (5.8)	6 (2.6)
**Previous biological therapy**, ***n*** **(%)**				
0	70 (73.7)	53 (76.8)	57 (82.6)	180 (77.3)
1	14 (14.7)	10 (14.5)	10 (14.5)	34 (14.6)
2+	11 (11.6)	6 (8.7)	2 (2.9)	19 (8.2)
**bDMARD at baseline**, ***n*** **(%)**	8 (8.4)	7 (10.1)	6 (8.7)	21 (9.0)
Discontinued	8 (8.4)	7 (10.1)	6 (8.7)	21 (9.0)
**csDMARD at baseline**, ***n*** **(%)**	85 (89.5)	10 (14.5)	39 (56.5)	134 (57.5)
Continued	30 (31.6)	5 (7.2)	21 (30.4)	56 (24.0)
Discontinued	50 (52.6)	5 (7.2)	17 (24.6)	72 (30.9)
Unknown	5 (5.3)	0 (0.0)	1 (1.4)	6 (2.6)
**tsDMARD at baseline**, ***n*** **(%)**	0 (0.0)	0 (0.0)	3 (4.3)	3 (1.3)
Discontinued	0 (0.0)	0 (0.0)	3 (4.3)	3 (1.3)
**Glucocorticoids at baseline**, ***n*** **(%)**	12 (12.6)	7 (10.1)	5 (7.2)	24 (10.3)
Continued	3 (3.2)	2 (2.9)	3 (4.3)	8 (3.4)
Discontinued	9 (9.5)	5 (7.2)	2 (2.9)	16 (6.9)

*axSpA, axial spondyloarthritis; bDMARD, biologic disease-modifying anti-rheumatic drug; BMI, body mass index; csDMARD, conventional synthetic disease-modifying anti-rheumatic drug; PsA, psoriatic arthritis; Q1, first quartile; Q3, third quartile; RA, rheumatoid arthritis; SD, standard deviation; tsDMARD, targeted synthetic disease-modifying antirheumatic drug*.

The reason for golimumab discontinuation was most often lack of efficacy (*n* = 7), followed by side effects (*n* = 4), remission (*n* = 3), other reasons/unknown (*n* = 3) and switch to another treatment (*n* = 2). The majority of patients initiating golimumab were naïve to biological therapy (*n* = 180, 77.3%). Out of the 53 biologics experienced patients (22.7%), 34 patients received 1 prior biological agent. At baseline visit, 21 patients (9%) were still on biologic disease-modifying anti-rheumatic drugs (bDMARDs) and switched to golimumab. The remaining 32 patients discontinued their bDMARDs prior to study start; among them 13 (5.6%) were on csDMARDs, 2 (0.9%) were on glucocorticoids, and another 2 were on csDMARDs plus glucocorticoids at baseline visit. Furthermore, 134 patients (57.5%) were using csDMARDs at baseline and 24 patients (10.3%) were on glucocorticoids; these drugs were continued during the golimumab therapy by 56 (24%) and eight patients 8 (3.4%), respectively (Table 1). Additionally, three patients (1.3%) were on targeted synthetic DMARDs which were discontinued at baseline visit.

Disease activity decreased in all groups after start of golimumab (Supplementary Tables 1, 2): CDAI in RA decreased in comparison to baseline by −18.4 (± 12.7, *p* < 0.0001) and in PsA by −15.2 (± 10.0, *p* < 0.0001) at 3 months and by −24.3 (± 13.5, *p* < 0.0001) and −21.7 (± 8.5, *p* < 0.0001) at 24 months, respectively. BASDAI decreased by −2.7 (± 2.4, *p* < 0.0001) at 3 months and by –4.4 (± 2.1, *p* < 0.0001) at 24 months.

### Improvement of Work Productivity and Daily Activity Impairment by Golimumab

**Figure 2:**
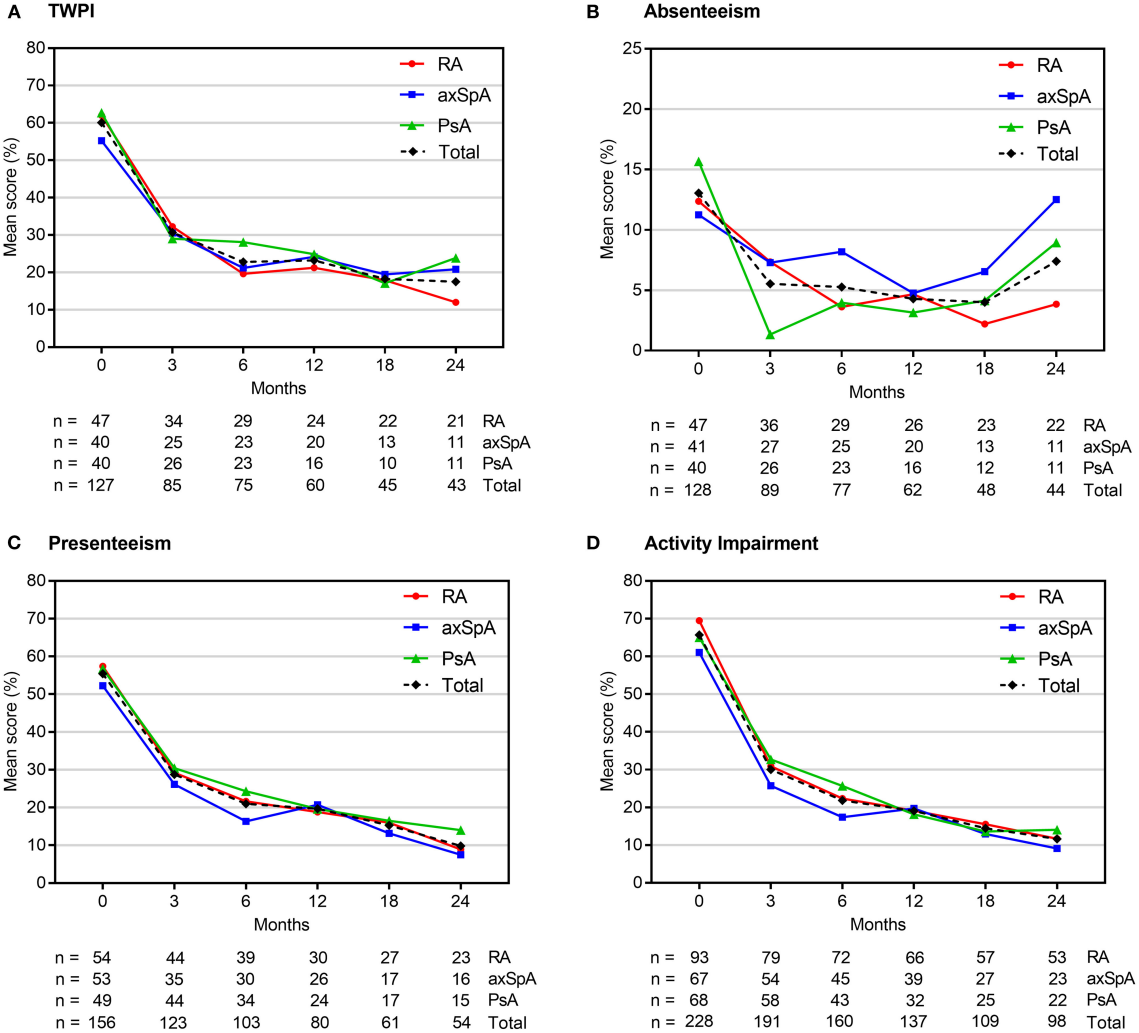
Mean WPAI scores at each study visit. Scores are expressed as percentages (from 0 to 100%), with higher values indicating a greater impairment. axSpA, axial spondyloarthritis; PsA, psoriatic arthritis; RA, rheumatoid arthritis; TWPI, total work productivity impairment; WPAI, work productivity and daily activity impairment.

Individual WPAI scores at each study visit are shown in Figure 2, Supplementary Table 3. Scores changed most in the first 3 months of therapy; in subsequent visits, there was a continuous decline, albeit the delta between the visits became continuously smaller. At 3 months, we observed a reduction (indicating an improvement) of the mean TWPI score in all employed patients (*n* = 85, pooled analysis of RA, axSpA, PsA) by −33.9% (± 30.8%; *p* < 0.0001). Similarly, mean activity impairment scores (*n* = 191) decreased at month 3 by 37.2% (± 29.9%; *p* < 0.0001), presenteeism scores by −28.9% (± 31.4%; *p* < 0.0001), and absenteeism scores by −6.9% (± 22.2%; *p* < 0.0001). Between baseline and month 24, mean TWPI scores decreased by −46.4% (± 34.5%; *p* < 0.0001), presenteeism scores by −51.3% (± 28.4%; *p* < 0.0001) and activity impairment scores by −59.9% (± 24.8%; *p* < 0.0001). The reduction in mean absenteeism score at month 24 was not statistically significant (– 4.6 ± 36.7%; *p* = 0.1918).

Individual analyses of RA, axSpA and PsA patients revealed a similar statistically significant improvements in TWPI, presenteeism and activity impairment scores at 3 months in all groups (mean change range: −32.5 to −34.8%; −25.1 to −33%; and −32.6 to −40.5%, see Supplementary Table 3). Statistically significant decreases in the mean absenteeism scores were observed only in axSpA and PsA patients (by −9.7 and −7%, respectively). TWPI, presenteeism and activity impairment scores continued to improve up to month 24 in all patient groups (mean change range: −33.6 to −58.3%; −31.2 to −61.7%; and −36.3 to −62.6%, respectively), whereas the absenteeism scores statistically significantly decreased in the RA population (mean change range: −7.5 to 10.5%, respectively) but not in axSpA and PsA patients (Supplementary Table 3). The number of cases evaluated at this time point, however, was relatively small (*n* = 43 for TWPI; *n* = 44 for absenteeism, *n* = 54 for presenteeism and *n* = 98 for activity impairment).

Presenteeism and activity impairment scores decreased throughout the study in both biologics naïve patients (pooled RA, axSpA and PsA) and in those with ≥1 prior biological therapy (Supplementary Table 4). Statistically significant reductions in TWPI and absenteeism scores were observed mostly in biologics naïve patients. In biologics experienced patients, TWPI scores decreased from baseline only at month 3, whereas absenteeism scores did not change throughout the study. The magnitude of WPAI scores change from baseline was not statistically significantly different between biologics naïve and biologics experienced patients.

Overall, proportion of missed working days did not change from the 6 months prior to baseline compared to the first and second year after start of golimumab (data not shown). In total, 65.7% of patients (*n* = 153/233) were employed and 34.3% (*n* = 80/233) were not employed at baseline. These numbers did not change significantly after treatment start with golimumab (data not shown). Five unemployed patients (6.3%) took up paid job throughout the study.

### Quality of Life Analysis

QoL scores decreased (indicating an improvement) in all 3 disease groups from baseline throughout month 24 (Supplementary Table 10). Mean RAQoL score was 16 (± 8) at baseline, 7.4 (± 6.4) at month 3 and 3.2 (± 3.8) at month 24. Corresponding values for the ASQoL were 9.2 (± 4.3), 4.1 (± 4.4) 1.2 (± 2.3), respectively and for the PsAQoL 11 (± 5.4), 6.0 (± 5.5) and 2.8 (± 5.1). Decrease in RAQoL, ASQoL and PsAQoL scores from the baseline was statistically significant at each study visit (*p* < 0.0001).

### Factors Associated With Work Productivity/Daily Activity Impairment

Univariate linear regression analysis detected a positive association between WPAI subscores and CDAI and BASDAI scores at baseline in RA (but not PsA) and axSpA patients, respectively (see Supplementary Tables 5–7 for details). Each single point increase in baseline CDAI in RA patients for example translated into a 1.1% increase in TWPI subscore. CDAI (RA) and BASDAI (axSpA) correlated with TWPI subscore at each visit until month 12, and they were associated with presenteeism and activity impairment subscores at each time-point throughout the entire study period. The association of CDAI and BASDAI with absenteeism was present only at baseline. In PsA, the linkage between CDAI scores and TWPI, presenteeism and activity impairment emerged at month 3 and was then maintained throughout the study. Absenteeism subscores in PsA patients associated with CDAI only at month 24.

HRQoL was associated with TWPI (during the first 12 months of therapy) and with activity impairment (see Supplementary Tables 8, 9). Further associations were detected between prior biologic treatment and TWPI score at month 24, age and activity impairment score at month 3 and 24, and employment status at baseline and activity impairment score at month 3.

### Safety Analysis

There were 138 TEAEs (18 serious) which occurred in 107 patients (45.9%), with 47 TEAEs in 43 patients (18.5%) considered to be drug-related. The most frequently reported drug-related TEAEs included: drug ineffectiveness (38.3%, *n* = 18) and off label use (8.5%, *n* = 4).

## Discussion

Our study demonstrated improvement in WPAI, QoL and disease activity in Austrian RA, PsA and axSpA patients treated with golimumab in real-world conditions. We observed improvements in all WPAI subscores (except for absenteeism in RA patients, see below) already during the first 3 months of the study. Interestingly, changes in presenteeism and activity impairment subscores were observed only in patients who were naïve to biologics. This suggests that golimumab has a rapid mode of action particularly in less heavily pretreated patients, who are less likely to suffer from advanced disease. Scores for TWPI, presenteeism and activity impairment in all patient groups, and absenteeism scores in RA patients continued to improve until month 24.

Compared with previous real-world evidence on golimumab from the non- interventional GO-ART study in Germany, the present study demonstrated more pronounced improvements in TWPI and activity impairment in RA, axSpA and PsA patients (19). Our study also demonstrated higher improvements in QoL at month 3 and 24 than the GO-ART study (19). These differences may be the result of a higher proportion of biologic naive patients in this study compared to the GO-ART study (77.3 vs. 67.5%).

Golimumab therapy markedly reduced the disease activity in each indication. These results are in line with the data from other observational studies investigating golimumab (18, 30). Univariate linear regression analysis demonstrated that the impact of disease activity on work productivity and work activity differed between the indications: a higher disease activity was associated with a greater impairment in work productivity and activity in RA and axSpA whereas this association was less evident for PsA. In RA and axSpA, we observed a linkage of disease activity with TWPI (during the first year of therapy), presenteeism and activity impairment (throughout the entire study period) while the association with absenteeism was lost after baseline. In contrast, the association between CDAI and WPAI subscores in PsA patients appeared only after the start of golimumab therapy and it gradually increased during the treatment. Potentially, this difference could be due to the fact that besides joint inflammation, PsA patients frequently suffer from enthesitis, dactylitis, axial and nail problems. CDAI questionnaire however focuses mainly on the arthritis component and it does not capture the psoriasis component of the disease, which could also contribute to the work impairment. In fact, Mease et al. have recently shown that PsA patients with enthesitis had worse work impairment than those without (31). Several studies investigated the association between WPAI scores and disease activity in patients treated with other biologics (26, 32, 33). For instance, TWPI and activity impairment were shown to correlate with BASDAI and CDAI scores in axSpA and PsA patients treated with adalimumab (26, 33), however, these findings were reported only for baseline or the first 24 weeks of therapy. In contrast, our results suggest that reductions in disease activity over 2 years of therapy with golimumab result in sustained improvement in work productivity and ability to perform daily activities.

RA, axSpA and PsA are associated with progressive disability causing work restrictions and lost work productivity and, in turn, diminished participation in the labor market (34). Patients suffering from rheumatic diseases are less likely to maintain their paid work or to take new work opportunities. These restrictions affect both personal finances and overall economy (35). In our study, only 6.3% of unemployed patients at baseline took up a paid job during the study. These data indicate that golimumab may have only a limited capability to reintegrate unemployed patients into the labor market, although these results should be interpreted with caution given the low number of unemployed patients in our study (80 patients) and the multiple other factors that determine whether or not people may or want to find a job. Furthermore, we found that improvements in TWPI induced by golimumab were mainly driven by gains in presenteeism, although a positive impact on absenteeism was also seen during the first 3 months of therapy in axSpA and PsA patients. On the contrary, absenteeism in RA patients improved after 6 months of treatment indicating a long-term effect of golimumab on reduction of the number of missed working days in that group. Nevertheless, it should be noted that the proportion of unemployed (including retired and incapacitated for work) in the RA group was high at 48.4%.

Although these data are in line with other reports indicating that 33.3 to 54.0% of RA patients in Germany are unemployed for any reason (18, 19), such a high proportion of unemployed patients could limit the power to detect the impact of golimumab on absenteeism in our study.

Overall, the safety profile was comparable to the previous reports (18); no new safety concerns have been identified for golimumab.

This NIS has several limitations. First, 2-year observation period may not have been sufficiently long to capture long-term therapy outcomes in the chronic diseases investigated. Second, due to a single-arm design, the comparison between patients treated with golimumab and those treated with other medications was not possible. Third, radiographic data was not collected within this study, hence, the impact of structural joint damage on WPAI could not be assessed. Similarly, we did not analyze clinical deformity data which could provide an interesting insight into the factors affecting the activity impairment. Fourth, some patients in this study were using csDMARDs and glucocorticoids for at least some time together with golimumab.

Potentially, this could affect the outcomes, however, only approximately a quarter of patients received such combined treatment and therefore its impact on the overall study findings was rather limited. Fifth, since the patients were followed for up to 2 years or until they discontinued golimumab, we could not compare the outcomes between patients who continued and those who stopped golimumab treatment.

## Conclusions

This prospective non-interventional, open-label study in Austria supported a beneficial effect of golimumab on WPAI and QoL, and decreasing disease activity in patients with RA, PsA and axSpA in a routine clinical setting. Improvements in WPAI, QoL and disease activity were observed early during therapy and maintained over the 24 months study period. Furthermore, gains in TWPI, presenteeism and activity impairment, and to a lesser extent in absenteeism, were associated with reduction in disease activity.

## Data Availability Statement

The original contributions presented in the study are included in the article/Supplementary Material, further inquiries can be directed to the corresponding author/s.

## Ethics Statement

The study involving human participants were reviewed and approved by the Leading Ethics Committee at the Medical University Graz and the Ethics Committee of the Federal State of Carinthia (approval number: 28-312 ex 15/16). The patients/participants provided their written informed consent to participate in this study.

## Author Contributions

CD principle investigator, contributed to conception and design of the study, manuscript revision, read, and approved the submitted version. GN is an employee of the company MSD Austria, supervision, conception and design of the study, Project administration, manuscript revision, read, and approved the submitted version. TM, OZ, UK, SE, JR-P, AT, BY-B, TS, PP, and AK contributed to data acquisition, manuscript revision, read, and approved the submitted version. This study including writing service for the manuscript was funded by MSD Austria.

## Funding

This study received funding from MSD Austria. The funder had the following involvement with the study: study design, framework for the data collection and analysis, decision to publish and preparation of the manuscript.

## Conflict of Interest

This study received funding from MSD Austria. The funder had the following involvement with the study: study design, framework for the data collection and analysis, decision to publish and preparation of the manuscript. The remaining authors declare that the research was conducted in the absence of any commercial relationship. The investigators received honoraria for the time spent examining and documenting patients.

## Publisher's Note

All claims expressed in this article are solely those of the authors and do not necessarily represent those of their affiliated organizations, or those of the publisher, the editors and the reviewers. Any product that may be evaluated in this article, or claim that may be made by its manufacturer, is not guaranteed or endorsed by the publisher.
