# Supplementary material for: A Prospective Study to Evaluate the Impact of Golimumab Therapy on Work Productivity and Activity, and Quality of Life in Patients With Rheumatoid Arthritis, Psoriasis Arthritis and Axial Spondyloarthritis in a Real Life Setting in AUSTRIA. The GO-ACTIVE Study
Source: Front Med (Lausanne). 2022 Jun 2;9:881943. doi: 10.3389/fmed.2022.881943 (PMC9201205; doi:10.3389/fmed.2022.881943)
Supplement: Supplementary file 1 [file Data_Sheet_1.doc]

# Supplementary file 1

**Supplementary Tables**

Supplementary Table 1. Course of clinical disease activity markers in RA and PsA patients

|  | **RA** | | **PsA** | |
| --- | --- | --- | --- | --- |
|  | **N** | **Mean (SD)** | **N** | **Mean (SD)** |
| **PGA** |  |  |  |  |
| Baseline | 95 | 7.0 (2.2) | 69 | 6.7 (1.7) |
| Month 3 | 87 | 3.5 (2.2) | 61 | 3.2 (2.4) |
| Month 6 | 76 | 2.7 (1.9) | 46 | 2.7 (2.1) |
| Month 12 | 70 | 2.4 (2.3) | 37 | 2.0 (2.2) |
| Month 18 | 63 | 1.9 (1.4) | 31 | 1.8 (2.0) |
| Month 24 | 59 | 1.9 (2.0) | 26 | 1.5 (1.8) |
| **SJC** |  |  |  |  |
| Baseline | 95 | 6.3 (4.4) | 69 | 4.6 (5.1) |
| Month 3 | 87 | 1.8 (2.8) | 61 | 0.5 (1.1) |
| Month 6 | 76 | 1.0 (2.4) | 46 | 0.5 (1.7) |
| Month 12 | 70 | 0.6 (1.5) | 37 | 0.4 (1.0) |
| Month 18 | 63 | 0.6 (1.6) | 31 | 0.2 (0.7) |
| Month 24 | 59 | 0.3 (0.8) | 26 | 0.2 (0.5) |
| **TJC** |  |  |  |  |
| Baseline | 95 | 9.2 (6.3) | 69 | 6.3 (4.8) |
| Month 3 | 87 | 2.9 (4.1) | 61 | 1.9 (2.8) |
| Month 6 | 76 | 1.4 (3.3) | 46 | 1.4 (2.2) |
| Month 12 | 70 | 1.2 (2.4) | 37 | 1.2 (2.3) |
| Month 18 | 63 | 0.9 (1.6) | 31 | 0.8 (2.0) |
| Month 24 | 59 | 0.9 (1.6) | 26 | 0.5 (1.1) |
| **EGA** |  |  |  |  |
| Baseline | 95 | 6.0 (2.0) | 69 | 5.8 (1.7) |
| Month 3 | 87 | 2.4 (2.0) | 61 | 2.2 (1.9) |
| Month 6 | 76 | 1.6 (1.6) | 46 | 1.9 (1.8) |
| Month 12 | 70 | 1.6 (1.9) | 37 | 1.2 (1.5) |
| Month 18 | 63 | 1.1 (1.1) | 31 | 0.9 (1.2) |
| Month 24 | 59 | 0.9 (0.9) | 26 | 0.6 (0.8) |
| **CDAI** |  |  |  |  |
| Baseline | 95 | 28.5 (12.3) | 69 | 23.4 (9.6) |
| Month 3 | 87 | 10.6 (9.5) | 61 | 7.7 (6.6) |
| Month 6 | 76 | 6.7 (8.1) | 46 | 6.5 (6.4) |
| Month 12 | 70 | 5.8 (7.0) | 37 | 4.8 (6.0) |
| Month 18 | 63 | 4.4 (4.6) | 31 | 3.7 (4.5) |
| Month 24 | 59 | 4.1 (4.3) | 26 | 2.8 (3.2) |

CDAI is a numerical sum of EGA + PGA + SJC + TJC with score ≤2.8 indicating disease remission, 2.8 to 10 indicating a low disease activity, 10 to 22 indicating a moderate disease activity, and >22 indicating a high disease activity. EGA and PGA were measured on a 10 cm visual analogue scale with 0=best and 10=worst. SJC and TJC are based on a 28-joint assessment. CDAI, Clinical Disease Activity Index; EGA, Evaluator's Global Assessment; PGA, Patient Global disease Activity estimate; PsA, psoriatic arthritis; RA, rheumatoid arthritis; SD, standard deviation; SJC, Swollen Joint Count; TJC, Tender Joint Count.

Supplementary Table 2. BASDAI scores at each study visit in axSpA patients

| **BASDAI score** | **N** | **Mean (SD)** |
| --- | --- | --- |
| **Tiredness and exhaustion** |  |  |
| Baseline | 66 | 6.2 (2.4) |
| Month 3 | 61 | 3.4 (2.3) |
| Month 6 | 51 | 2.7 (2.6) |
| Month 12 | 40 | 2.3 (2.5) |
| Month 18 | 29 | 1.4 (1.3) |
| Month 24 | 25 | 1.8 (1.7) |
| **Neck-, back- or hip- pain** |  |  |
| Baseline | 66 | 6.6 (2.5) |
| Month 3 | 61 | 3.2 (2.8) |
| Month 6 | 51 | 2.3 (2.3) |
| Month 12 | 40 | 2.0 (2.7) |
| Month 18 | 29 | 1.4 (1.9) |
| Month 24 | 25 | 1.2 (1.6) |
| **Pain/ swelling in other joints** |  |  |
| Baseline | 66 | 4.8 (3.1) |
| Month 3 | 61 | 2.5 (2.6) |
| Month 6 | 51 | 1.5 (1.6) |
| Month 12 | 40 | 1.7 (2.3) |
| Month 18 | 29 | 0.9 (1.4) |
| Month 24 | 25 | 0.9 (1.3) |
| **Complaints to touch or pressure** |  |  |
| Baseline | 66 | 4.7 (2.8) |
| Month 3 | 61 | 2.3 (2.4) |
| Month 6 | 51 | 1.6 (2.0) |
| Month 12 | 40 | 1.6 (2.2) |
| Month 18 | 29 | 1.0 (1.2) |
| Month 24 | 25 | 0.8 (1.1) |
| **Severity of morning stiffness** |  |  |
| Baseline | 66 | 5.8 (2.7) |
| Month 3 | 61 | 2.7 (2.6) |
| Month 6 | 51 | 1.9 (2.0) |
| Month 12 | 40 | 1.6 (2.1) |
| Month 18 | 29 | 1.2 (1.6) |
| Month 24 | 25 | 1.0 (1.3) |
| **Duration of morning stiffness** |  |  |
| Baseline | 66 | 5.2 (3.1) |
| Month 3 | 61 | 1.6 (1.8) |
| Month 6 | 51 | 1.4 (1.7) |
| Month 12 | 40 | 1.3 (1.8) |
| Month 18 | 29 | 0.9 (1.5) |
| Month 24 | 25 | 0.8 (0.8) |
| **BASDAI** |  |  |
| Baseline | 66 | 5.6 (2.1) |
| Month 3 | 61 | 2.7 (2.2) |
| Month 6 | 51 | 1.9 (1.7) |
| Month 12 | 40 | 1.8 (2.2) |
| Month 18 | 29 | 1.1 (1.2) |
| Month 24 | 25 | 1.1 (1.2) |

Patient’s response to each of the BASDAI questions was measured on a 10 cm visual analogue scale with 0=no complaints and 10=unbearable pain. The final BASDAI score is the sum of the scores for questions “Tiredness and exhaustion”, “Neck-, back- or hip- pain”, “Pain/ swelling in other joints” and “Complaints to touch or pressure”, and the average of questions “Severity of morning stiffness” and “Duration of morning stiffness”; the resulting value was the divided by 5. The final BASDAI score ranges between 0 and 10 with a higher score indicating a greater degree of disease activity. axSpA, axial spondyloarthritis; BASDAI, Bath Ankylosing Spondylitis Disease Activity Index; SD, standard deviation.

Supplementary Table 3. WPAI scores in RA, axSpA and PsA patients each study visit. Scores are expressed as percentages (from 0% to 100%), with higher values indicating a greater impairment.

| **WPAI score** | **RA** | | | **axSpA** | | | **PsA** | | | **Total** | | |
| --- | --- | --- | --- | --- | --- | --- | --- | --- | --- | --- | --- | --- |
|  | **N** | **Mean (SD)** | **p-Value** | **N** | **Mean (SD)** | **p-Value** | **N** | **Mean (SD)** | **p-Value** | **N** | **Mean (SD)** | **p-Value** |
| **TWPI** | | | | | | | | | | | | |
| Baseline | 47 | 62.0 (28.0) |  | 40 | 55.2 (29.1) |  | 40 | 62.7 (25.1) |  | 127 | 60.1 (27.5) |  |
| Month 3 | 34 | 32.3 (30.4) | **<0.0001** | 25 | 30.5 (29.1) | **<0.0001** | 26 | 29.0 (26.7) | **<0.0001** | 85 | 30.7 (28.6) | **<0.0001** |
| Month 6 | 29 | 19.6 (18.1) | **<0.0001** | 23 | 21.2 (26.3) | **<0.0001** | 23 | 28.1 (25.8) | **<0.0001** | 75 | 22.8 (23.5) | **<0.0001** |
| Month 12 | 24 | 21.2 (22.6) | **<0.0001** | 20 | 24.2 (27.8) | **<0.0001** | 16 | 24.9 (24.3) | **0.0017** | 60 | 23.2 (24.5) | **<0.0001** |
| Month 18 | 22 | 17.9 (20.7) | **<0.0001** | 13 | 19.5 (26.1) | **0.0032** | 10 | 17.1 (24.3) | **0.0117** | 45 | 18.3 (22.9) | **<0.0001** |
| Month 24 | 21 | 12.0 (13.6) | **<0.0001** | 11 | 20.8 (28.8) | **0.0117** | 11 | 23.9 (31.7) | **0.0186** | 43 | 17.5 (23.9) | **<0.0001** |
| **Absenteeism** | | | | | | | | | | | | |
| Baseline | 47 | 12.4 (26.5) |  | 41 | 11.2 (24.7) |  | 40 | 15.7 (29.0) |  | 128 | 13.0 (26.6) |  |
| Month 3 | 36 | 7.3 (21.3) | 0.0785 | 27 | 7.3 (24.7) | **0.0078** | 26 | 1.3 (6.5) | **0.0117** | 89 | 5.5 (19.5) | **<0.0001** |
| Month 6 | 29 | 3.6 (12.6) | **0.0244** | 23 | 8.2 (22.2) | 0.3594 | 23 | 4.0 (12.6) | 0.1563 | 75 | 5.3 (16.4) | **0.0052** |
| Month 12 | 26 | 4.7 (14.8) | 0.1484 | 20 | 4.8 (21.3) | 0.0645 | 16 | 3.2 (10.4) | 0.6875 | 62 | 4.3 (16.0) | **0.0204** |
| Month 18 | 23 | 2.2 (10.0) | **0.0234** | 13 | 6.5 (24.2) | 0.875 | 12 | 4.1 (11.1) | 0.625 | 48 | 4.0 (15.8) | 0.107 |
| Month 24 | 22 | 3.8 (12.2) | **0.0234** | 11 | 12.5 (31.1) | 0.875 | 11 | 8.9 (27.5) | 0.75 | 44 | 7.4 (22.4) | 0.1918 |
| **Presenteeism** | | | | | | | | | | | | |
| Baseline | 54 | 57.4 (27.5) |  | 53 | 52.3 (29.1) |  | 49 | 56.7 (25.1) |  | 156 | 55.5 (27.3) |  |
| Month 3 | 44 | 29.2 (24.4) | **<0.0001** | 35 | 26.2 (27.2) | **<0.0001** | 44 | 30.4 (26.3) | **<0.0001** | 123 | 28.8 (25.7) | **<0.0001** |
| Month 6 | 39 | 21.6 (20.4) | **<0.0001** | 30 | 16.4 (19.3) | **<0.0001** | 34 | 24.3 (24.8) | **<0.0001** | 103 | 21.0 (21.7) | **<0.0001** |
| Month 12 | 30 | 18.8 (19.4) | **<0.0001** | 26 | 20.7 (25.5) | **<0.0001** | 24 | 19.6 (20.7) | **<0.0001** | 80 | 19.7 (21.6) | **<0.0001** |
| Month 18 | 27 | 16 (17.9) | **<0.0001** | 17 | 13.2 (16.4) | **<0.0001** | 17 | 16.5 (21.2) | **0.0006** | 61 | 15.3 (18.2) | **<0.0001** |
| Month 24 | 23 | 8.9 (10.1) | **<0.0001** | 16 | 7.5 (8.6) | **0.0001** | 15 | 14 (20.6) | **0.0007** | 54 | 9.8 (13.3) | **<0.0001** |
| **Activity impairment** | | | | | | | | | | | | |
| Baseline | 93 | 69.5 (25.4) |  | 67 | 61.0 (26.4) |  | 68 | 65.0 (22.2) |  | 228 | 65.7 (24.9) |  |
| Month 3 | 79 | 30.9 (23.2) | **<0.0001** | 54 | 25.7 (24.2) | **<0.0001** | 58 | 32.7 (27.2) | **<0.0001** | 191 | 30.0 (24.8) | **<0.0001** |
| Month 6 | 72 | 22.3 (21.3) | **<0.0001** | 45 | 17.4 (18.9) | **<0.0001** | 43 | 25.7 (25.0) | **<0.0001** | 160 | 21.8 (21.8) | **<0.0001** |
| Month 12 | 66 | 19.1 (19.8) | **<0.0001** | 39 | 19.7 (23.8) | **<0.0001** | 32 | 18.2 (19.0) | **<0.0001** | 137 | 19.1 (20.7) | **<0.0001** |
| Month 18 | 57 | 15.5 (13.4) | **<0.0001** | 27 | 13.0 (13.8) | **<0.0001** | 25 | 13.6 (17.5) | **<0.0001** | 109 | 14.5 (14.4) | **<0.0001** |
| Month 24 | 53 | 11.7 (11.6) | **<0.0001** | 23 | 9.1 (10.8) | **<0.0001** | 22 | 14.1 (19.7) | **<0.0001** | 98 | 11.6 (13.6) | **<0.0001** |

p-values are given for comparison with baseline. axSpA, axial spondyloarthritis; PsA, psoriatic arthritis; RA, rheumatoid arthritis; SD, standard deviation; TWPI, total work productivity impairment; WPAI, work productivity and daily activity impairment.

Supplementary Table 4. WPAI scores in biologics naïve and biologics experienced patients at each study visit. Scores are expressed as percentages (from 0% to 100%), with higher values indicating a greater impairment.

| **WPAI score** | **Biologics naïve** | | | **Biologics experienced** | | | **Total** | | |
| --- | --- | --- | --- | --- | --- | --- | --- | --- | --- |
|  | **N** | **Mean (SD)** | **p-Value** | **N** | **Mean (SD)** | **p-Value** | **N** | **Mean (SD)** | **p-Value** |
| **TWPI** | | | | | | | | | |
| Baseline | 101 | 59.2 (27.0) |  | 26 | 63.5 (29.6) |  | 127 | 60.1 (27.5) |  |
| Month 3 | 86 | 29.8 (28.9) | **<0.0001** | 17 | 35.2 (27.6) | **0.002** | 85 | 30.7 (28.6) | **<0.0001** |
| Month 6 | 74 | 20.9 (21.6) | **<0.0001** | 11 | 35.9 (31.7) | 0.127 | 75 | 22.8 (23.5) | **<0.0001** |
| Month 12 | 61 | 22.0 (23.1) | **<0.0001** | 7 | 33.6 (35.4) | 0.1094 | 60 | 23.2 (24.5) | **<0.0001** |
| Month 18 | 44 | 16.9 (19.7) | **<0.0001** | 8 | 25.6 (36.8) | 0.0547 | 45 | 18.3 (22.9) | **<0.0001** |
| Month 24 | 39 | 13.4 (16.3) | **<0.0001** | 8 | 37.5 (42.0) | 0.0859 | 43 | 17.5 (23.9) | **<0.0001** |
| **Absenteeism** | | | | | | | | | |
| Baseline | 102 | 12.7 (26.3) |  | 26 | 14.3 (28.2) |  | 128 | 13.0 (26.6) |  |
| Month 3 | 90 | 6.0 (20.7) | **0.0004** | 17 | 3.2 (12.1) | 0.0625 | 89 | 5.5 (19.5) | **<0.0001** |
| Month 6 | 75 | 3.4 (11.3) | **0.0005** | 12 | 16.7 (32.6) | 0.5 | 75 | 5.3 (16.4) | **0.0052** |
| Month 12 | 62 | 2.4 (9.9) | **0.0083** | 8 | 18.8 (37.2) | 1.0 | 62 | 4.3 (16.0) | **0.0204** |
| Month 18 | 46 | 1.4 (6.2) | **0.0494** | 9 | 17.3 (35.1) | 1.0 | 48 | 4.0 (15.8) | 0.107 |
| Month 24 | 40 | 2.6 (9.4) | **0.0458** | 8 | 31.3 (45.8) | 1.0 | 44 | 7.4 (22.4) | 0.1918 |
| **Presenteeism** | | | | | | | | | |
| Baseline | 125 | 54.5 (27.4) |  | 31 | 59.4 (27.0) |  | 156 | 55.5 (27.3) |  |
| Month 3 | 110 | 28.4 (26.0) | **<0.0001** | 27 | 30.4 (25.2) | **0.0003** | 123 | 28.8 (25.7) | **<0.0001** |
| Month 6 | 95 | 20.1 (22.0) | **<0.0001** | 18 | 25.6 (19.8) | **0.0097** | 103 | 21.0 (21.7) | **<0.0001** |
| Month 12 | 76 | 21.3 (22.2) | **<0.0001** | 11 | 8.2 (13.3) | **0.001** | 80 | 19.7 (21.6) | **<0.0001** |
| Month 18 | 56 | 17.0 (18.6) | **<0.0001** | 10 | 6.0 (12.7) | **0.002** | 61 | 15.3 (18.2) | **<0.0001** |
| Month 24 | 47 | 10.9 (14.0) | **<0.0001** | 11 | 5.5 (9.3) | **0.001** | 54 | 9.8 (13.3) | **<0.0001** |
| **Activity impairment** | | | | | | | | | |
| Baseline | 176 | 65.5 (23.9) |  | 52 | 66.2 (28.5) |  | 228 | 65.7 (24.9) |  |
| Month 3 | 152 | 29.7 (24.7) | **<0.0001** | 41 | 31.0 (25.2) | **<0.0001** | 191 | 30.0 (24.8) | **<0.0001** |
| Month 6 | 131 | 21.7 (22.8) | **<0.0001** | 32 | 22.5 (17.8) | **<0.0001** | 160 | 21.8 (21.8) | **<0.0001** |
| Month 12 | 112 | 19.8 (22.0) | **<0.0001** | 27 | 15.9 (13.7) | **<0.0001** | 137 | 19.1 (20.7) | **<0.0001** |
| Month 18 | 87 | 14.9 (14.8) | **<0.0001** | 23 | 12.6 (13.2) | **<0.0001** | 109 | 14.5 (14.4) | **<0.0001** |
| Month 24 | 75 | 12.0 (13.9) | **<0.0001** | 23 | 10.4 (13.0) | **<0.0001** | 98 | 11.6 (13.6) | **<0.0001** |

p-values are given for comparison with baseline. SD, standard deviation; TWPI, total work productivity impairment; WPAI, work productivity and daily activity impairment.

Supplementary Table 5. Univariate linear regression analysis for association between CDAI scores and WPAI scores in RA patients at different time points.

| **Variable (CDAI score)** | **TWPI** | | | **Absenteeism** | | | **Presenteeism** | | | **Activity impairment** | | |
| --- | --- | --- | --- | --- | --- | --- | --- | --- | --- | --- | --- | --- |
|  | **Estimate of intercept** | **Estimate (SE)** | **p-Value** | **Estimate of intercept** | **Estimate (SE)** | **p-Value** | **Estimate of intercept** | **Estimate (SE)** | **p-Value** | **Estimate of intercept** | **Estimate (SE)** | **p-Value** |
| **Baseline** |  |  |  |  |  |  |  |  |  |  |  |  |
| PGA | 6.8 | 8.5 (1.3) | **<0.0001** | -8.5 | 3.2 (1.7) | 0.0632 | 2.3 | 8.7 (1.2) | **<0.0001** | 11.0 | 8.3 (0.9) | **<0.0001** |
| SJC | 46.3 | 2.5 (0.7) | **0.0007** | -8.0 | 3.3 (0.6) | **<0.0001** | 44.2 | 2.1 (0.7) | **0.0031** | 61.2 | 1.3 (0.6) | **0.0277** |
| TJC | 46.4 | 1.8 (0.5) | **0.0009** | -6.0 | 2.1 (0.4) | **<0.0001** | 42.3 | 1.7 (0.5) | **0.0007** | 57.2 | 1.3 (0.4) | **0.0011** |
| EGA | 36.7 | 4.4 (1.9) | **0.0259** | -6.0 | 3.2 (1.9) | 0.0920 | 27.5 | 5.3 (1.8) | **0.0040** | 44.8 | 4.1 (1.3) | **0.0021** |
| CDAI | 32.6 | 1.1 (0.2) | **<0.0001** | -17.8 | 1.1 (0.2) | **<0.0001** | 29.0 | 1.1 (0.2) | **<0.0001** | 44.2 | 0.9 (0.2) | **<0.0001** |
| **Month 3** |  |  |  |  |  |  |  |  |  |  |  |  |
| PGA | 1.5 | 9.6 (1.4) | **<0.0001** | 3.4 | 1.3 (1.4) | 0.3850 | 0.4 | 9.0 (0.9) | **<0.0001** | 5.0 | 7.3 (0.9) | **<0.0001** |
| SJC | 23.3 | 4.6 (1.7) | **0.0113** | 6.4 | 0.5 (1.3) | 0.6833 | 20.8 | 4.7 (1.2) | **0.0003** | 22.5 | 4.5 (0.8) | **<0.0001** |
| TJC | 19.4 | 4.4 (0.9) | **<0.0001** | 5.3 | 0.7 (0.8) | 0.3690 | 17.8 | 4.1 (0.6) | **<0.0001** | 20.7 | 3.5 (0.5) | **<0.0001** |
| EGA | 14.3 | 7.4 (2.0) | **0.0008** | 3.8 | 1.5 (1.6) | 0.3443 | 11.9 | 7.2 (1.4) | **<0.0001** | 14.9 | 6.6 (1.1) | **<0.0001** |
| CDAI | 12.0 | 1.9 (0.4) | **<0.0001** | 4.3 | 0.3 (0.3) | 0.3723 | 10.2 | 1.9 (0.3) | **<0.0001** | 13.2 | 1.7 (0.2) | **<0.0001** |
| **Month 12** |  |  |  |  |  |  |  |  |  |  |  |  |
| PGA | 12.2 | 4.0 (1.7) | **0.0286** | 1.0 | 1.6 (1.2) | 0.1783 | 8.4 | 4.6 (1.2) | **0.0008** | 5.8 | 5.6 (0.8) | **<0.0001** |
| SJC | 17.3 | 26.7 (11.3) | **0.0256** | 3.7 | 4.7 (5.7) | 0.4131 | 15.2 | 24.8 (8.4) | **0.0060** | 14.5 | 7.9 (1.4) | **<0.0001** |
| TJC | 17.5 | 9.2 (5.4) | 0.0969 | 4.9 | -0.4 (2.2) | 0.8540 | 15.9 | 5.0 (2.5) | 0.0534 | 13.8 | 4.7 (0.8) | **<0.0001** |
| EGA | 13.7 | 5.5 (2.1) | **0.0152** | 1.2 | 2.5 (1.5) | 0.0937 | 11.4 | 5.7 (1.7) | **0.0017** | 8.2 | 7.2 (1.0) | **<0.0001** |
| CDAI | 12.3 | 2.2 (0.8) | **0.0140** | 1.7 | 0.7 (0.5) | 0.2179 | 9.4 | 2.2 (0.6) | **0.0006** | 7.8 | 2.0 (0.3) | **<0.0001** |
| **Month 24** |  |  |  |  |  |  |  |  |  |  |  |  |
| PGA | 7.8 | 2.7 (1.8) | 0.1423 | 7.1 | -2.3 (1.6) | 0.1681 | 0.4 | 5.5 (0.7) | **<0.0001** | 3.5 | 4.8 (0.8) | **<0.0001** |
| SJC | 12.1 | -2.1 (14.2) | 0.8860 | 4.0 | -4.0 (12.8) | 0.7565 | 8.4 | 6.6 (7.5) | 0.3856 | 9.4 | 7.2 (1.8) | **0.0001** |
| TJC | 10.0 | 3.3 (1.9) | 0.0985 | 4.4 | -1.1 (1.8) | 0.5668 | 6.7 | 4.2 (1.2) | **0.0019** | 7.3 | 4.6 (0.7) | **<0.0001** |
| EGA | 11.7 | 0.4 (4.7) | 0.9382 | 7.9 | -6.2 (3.9) | 0.1243 | 3.4 | 7.9 (2.6) | **0.0053** | 5.4 | 6.8 (1.6) | **<0.0001** |
| CDAI | 7.7 | 1.5 (0.9) | 0.1178 | 6.9 | -1.1 (0.8) | 0.1823 | 1.0 | 2.8 (0.4) | **<0.0001** | 3.3 | 2.1 (0.3) | **<0.0001** |

The effect of CDAI score on WPAI score was calculated using the following linear regression model: WPAI score = Estimate of intercept + Estimate × CDAI score. Estimate indicates the incremental percent change in WPAI subscores for each single point change in CDAI score. Positive and negative estimate values correspond to an increase (i.e. worsening) or decrease (i.e. improvement) in the WPAI subscore. EGA and PGA were measured on a 10 cm visual analogue scale with 0=best and 10=worst. SJC and TJC are based on a 28-joint assessment. CDAI is a numerical sum of EGA + PGA + SJC + TJC with score ≤2.8 indicating disease remission, 2.8 to 10 indicating a low disease activity, 10 to 22 indicating a moderate disease activity, and >22 indicating a high disease activity. CDAI, Clinical Disease Activity Index; EGA, Evaluator's Global Assessment; PGA, Patient Global disease Activity estimate; RA, rheumatoid arthritis; SD, standard deviation; SJC, Swollen Joint Count; TJC, Tender Joint Count; TWPI, total work productivity impairment; WPAI, work productivity and daily activity impairment.

Supplementary Table 6. Univariate linear regression analysis for association between BASDAI scores and WPAI scores in axSpA patients.

| **Variable (BASDAI score)** | **TWPI** | | | **Absenteeism** | | | **Presenteeism** | | | **Activity impairment** | | |
| --- | --- | --- | --- | --- | --- | --- | --- | --- | --- | --- | --- | --- |
|  | **Estimate of intercept** | **Estimate (SE)** | **p-Value** | **Estimate of intercept** | **Estimate (SE)** | **p-Value** | **Estimate of intercept** | **Estimate (SE)** | **p-Value** | **Estimate of intercept** | **Estimate (SE)** | **p-Value** |
| **Baseline** |  |  |  |  |  |  |  |  |  |  |  |  |
| Complaints to touch or pressure | 24.6 | 6.9 (1.3) | **<0.0001** | -6.9 | 4.1 (1.3) | **0.0045** | 23.0 | 6.3 (1.2) | **<0.0001** | 34.4 | 5.7 (0.9) | **<0.0001** |
| Duration of morning stiffness | 15.5 | 6.9 (1.0) | **<0.0001** | -3.3 | 2.6 (1.3) | **0.0460** | 17.5 | 6.5 (0.9) | **<0.0001** | 39.8 | 4.1 (0.9) | **<0.0001** |
| Neck-, back- or hip- pain | 4.5 | 7.7 (1.4) | **<0.0001** | 4.9 | 1.0 (1.6) | 0.5300 | -0.5 | 8.0 (1.1) | **<0.0001** | 26.0 | 5.4 (1.1) | **<0.0001** |
| Pain/ swelling in other joints | 29.2 | 5.7 (1.2) | **<0.0001** | -2.0 | 2.9 (1.2) | **0.0249** | 27.0 | 5.4 (1.1) | **<0.0001** | 37.9 | 4.8 (0.9) | **<0.0001** |
| Shaping of morning stiffness | 14.0 | 6.7 (1.3) | **<0.0001** | 3.6 | 1.3 (1.5) | 0.3881 | 13.2 | 6.7 (1.2) | **<0.0001** | 33.7 | 4.8 (1.0) | **<0.0001** |
| Tiredness and exhaustion | 13.9 | 6.7 (1.7) | **0.0004** | 1.5 | 1.6 (1.8) | 0.3612 | 12.4 | 6.4 (1.5) | **0.0001** | 30.5 | 4.9 (1.2) | **0.0002** |
| BASDAI | -2.4 | 10.3 (1.3) | **<0.0001** | -9.8 | 3.8 (1.8) | **0.0381** | -7.7 | 10.8 (1.2) | **<0.0001** | 14.9 | 8.3 (1.1) | **<0.0001** |
| **Month 3** |  |  |  |  |  |  |  |  |  |  |  |  |
| Complaints to touch or pressure | 9.0 | 9.7 (1.4) | **<0.0001** | -2.1 | 4.4 (1.7) | **0.0126** | 6.7 | 8.6 (1.0) | **<0.0001** | 8.0 | 7.8 (0.9) | **<0.0001** |
| Duration of morning stiffness | 20.3 | 6.3 (2.2) | **0.0072** | 2.1 | 3.1 (1.9) | 0.1156 | 13.4 | 8.1 (1.8) | **<0.0001** | 16.7 | 5.8 (1.7) | **0.0009** |
| Neck-, back- or hip- pain | 6.5 | 7.1 (1.1) | **<0.0001** | 0.5 | 2.0 (1.3) | 0.1374 | 0.8 | 7.6 (0.6) | **<0.0001** | 3.4 | 7.0 (0.6) | **<0.0001** |
| Pain/ swelling in other joints | 8.3 | 8.5 (1.4) | **<0.0001** | -0.2 | 2.9 (1.6) | 0.0836 | 4.8 | 8.2 (0.9) | **<0.0001** | 8.4 | 7.0 (0.8) | **<0.0001** |
| Shaping of morning stiffness | 15.2 | 5.8 (1.6) | **0.0008** | 3.6 | 1.4 (1.6) | 0.3770 | 7.3 | 7.1 (1.1) | **<0.0001** | 10.3 | 5.7 (1.0) | **<0.0001** |
| Tiredness and exhaustion | 4.9 | 7.5 (1.5) | **<0.0001** | 0.2 | 2.1 (1.7) | 0.2184 | -3.0 | 8.3 (1.1) | **<0.0001** | -0.1 | 7.8 (0.9) | **<0.0001** |
| BASDAI | 2.9 | 10.0 (1.4) | **<0.0001** | -2.0 | 3.4 (1.8) | 0.0657 | -2.5 | 10.4 (0.8) | **<0.0001** | 1.0 | 9.3 (0.8) | **<0.0001** |
| **Month 12** |  |  |  |  |  |  |  |  |  |  |  |  |
| Complaints to touch or pressure | 11.5 | 7.2 (2.2) | **0.0034** | 7.3 | -1.3 (2.1) | 0.5428 | 2.6 | 9.5 (0.8) | **<0.0001** | 4.8 | 9.4 (0.9) | **<0.0001** |
| Duration of morning stiffness | 11.4 | 7.8 (2.5) | **0.0059** | 7.5 | -1.5 (2.3) | 0.5161 | 4.7 | 10.5 (1.6) | **<0.0001** | 5.8 | 10.3 (1.6) | **<0.0001** |
| Neck-, back- or hip- pain | 11.3 | 6.1 (1.7) | **0.0018** | 7.2 | -1.0 (1.7) | 0.5484 | 2.1 | 7.6 (0.7) | **<0.0001** | 3.2 | 8.2 (0.5) | **<0.0001** |
| Pain/ swelling in other joints | 10.8 | 7.3 (2.2) | **0.0033** | 7.5 | -1.4 (2.1) | 0.5169 | 1.9 | 9.4 (0.7) | **<0.0001** | 4.3 | 8.8 (1.0) | **<0.0001** |
| Shaping of morning stiffness | 10.8 | 7.4 (2.1) | **0.0019** | 7.3 | -1.3 (2.0) | 0.5418 | 3.4 | 9.5 (1.0) | **<0.0001** | 4.9 | 9.3 (1.1) | **<0.0001** |
| Tiredness and exhaustion | 9.1 | 7.4 (2.2) | **0.0032** | 8.3 | -1.6 (2.1) | 0.4494 | -0.5 | 8.4 (1.1) | **<0.0001** | -0.5 | 9.7 (0.5) | **<0.0001** |
| BASDAI | 9.9 | 7.6 (2.1) | **0.0019** | 7.7 | -1.4 (2.1) | 0.5045 | -0.1 | 9.9 (0.7) | **<0.0001** | 1.7 | 10.1 (0.7) | **<0.0001** |
| **Month 24** |  |  |  |  |  |  |  |  |  |  |  |  |
| Complaints to touch or pressure | 47.3 | -23.5 (13.9) | 0.1338 | 44.7 | -32.0 (14.2) | 0.0586 | 1.5 | 9.1 (2.9) | **0.0094** | 5.1 | 6.2 (2.4) | **0.0199** |
| Duration of morning stiffness | 39.3 | -16.7 (14.3) | 0.2800 | 35.4 | -24.8 (15.0) | 0.1416 | 2.5 | 7.9 (3.3) | **0.0352** | 6.4 | 5.5 (3.6) | 0.1429 |
| Neck-, back- or hip- pain | 34.4 | -6.7 (8.4) | 0.4497 | 30.5 | -12.0 (8.8) | 0.2179 | 5.0 | 2.5 (1.3) | 0.0903 | 5.9 | 3.6 (1.4) | **0.0212** |
| Pain/ swelling in other joints | 31.9 | -7.4 (12.4) | 0.5692 | 27.3 | -15.3 (13.2) | 0.2857 | 4.6 | 4.1 (1.9) | **0.0478** | 5.4 | 5.6 (1.7) | **0.0044** |
| Shaping of morning stiffness | 36.2 | -9.8 (10.9) | 0.3977 | 33.7 | -17.4 (11.2) | 0.1656 | 2.5 | 5.6 (1.9) | **0.0140** | 4.0 | 6.7 (2.2) | **0.0066** |
| Tiredness and exhaustion | 39.8 | -7.4 (7.5) | 0.3550 | 38.5 | -12.4 (7.8) | 0.1550 | 0.4 | 4.6 (1.4) | **0.0088** | 1.2 | 5.6 (1.4) | **0.0008** |
| BASDAI | 43.9 | -16.1 (12.7) | 0.2456 | 45.3 | -26.7 (12.3) | 0.0661 | 0.3 | 7.0 (1.9) | **0.0039** | 2.1 | 7.5 (1.9) | **0.0011** |

The effect of BASDAI score on WPAI score was calculated using the following linear regression model: WPAI score = Estimate of intercept + Estimate × BASDAI score. Estimate indicates the incremental percent change in WPAI subscores for each single point change in BASDAI score. Positive and negative estimate values correspond to an increase (i.e. worsening) or decrease (i.e. improvement) in the WPAI subscore. Patient’s response to each of the BASDAI questions was measured on a 10 cm visual analogue scale with 0=no complaints and 10=unbearable pain. The final BASDAI score is a sum of the scores for questions “Tiredness and exhaustion”, “Neck-, back- or hip- pain”, “Pain/ swelling in other joints” and “Complaints to touch or pressure”, and the average of questions “Severity of morning stiffness” and “Duration of morning stiffness”; the resulting value was the divided by 5. The final BASDAI score ranges between 0 and 10 with a higher score indicating a greater degree of disease activity. axSpA, axial spondyloarthritis; Bath Ankylosing Spondylitis Disease Activity Index (BASDAI); SD, standard deviation; TWPI, total work productivity impairment; WPAI, work productivity and daily activity impairment.

Supplementary Table 7. Univariate linear regression analysis for association between CDAI scores and WPAI scores in PsA patients.

| **Variable (CDAI score)** | **TWPI** | | | **Absenteeism** | | | **Presenteeism** | | | **Activity impairment** | | |
| --- | --- | --- | --- | --- | --- | --- | --- | --- | --- | --- | --- | --- |
|  | **Estimate of intercept** | **Estimate (SE)** | **p-Value** | **Estimate of intercept** | **Estimate (SE)** | **p-Value** | **Estimate of intercept** | **Estimate (SE)** | **p-Value** | **Estimate of intercept** | **Estimate (SE)** | **p-Value** |
| **Baseline** |  |  |  |  |  |  |  |  |  |  |  |  |
| PGA | 13.8 | 7.1 (2.2) | **0.0025** | -10.1 | 3.7 (2.8) | 0.1878 | 4.8 | 7.8 (1.8) | **<0.0001** | 15.3 | 7.4 (1.3) | **<0.0001** |
| SJC | 60.4 | 0.6 (1.0) | 0.5282 | 17.0 | -0.3 (1.1) | 0.7626 | 54.9 | 0.5 (0.9) | 0.6045 | 64.0 | 0.2 (0.5) | 0.6931 |
| TJC | 61.7 | 0.2 (0.8) | 0.8389 | 19.5 | -0.7 (1.0) | 0.5008 | 53.7 | 0.5 (0.8) | 0.4704 | 61.1 | 0.6 (0.6) | 0.2703 |
| EGA | 29.9 | 5.5 (2.5) | **0.0338** | -4.4 | 3.4 (3.0) | 0.2698 | 20.8 | 6.2 (2.0) | **0.0028** | 44.2 | 3.6 (1.6) | **0.0274** |
| CDAI | 51.1 | 0.5 (0.4) | 0.2240 | 16.5 | 0.0 (0.5) | 0.9435 | 40.7 | 0.7 (0.4) | 0.0626 | 52.2 | 0.5 (0.3) | 0.0512 |
| **Month 3** |  |  |  |  |  |  |  |  |  |  |  |  |
| PGA | 10.4 | 5.6 (1.9) | **0.0051** | -0.6 | 0.6 (0.5) | 0.2629 | 13.9 | 5.4 (1.6) | **0.0017** | 13.7 | 5.9 (1.3) | **<0.0001** |
| SJC | 26.0 | 7.3 (4.6) | 0.1229 | 1.6 | -0.6 (1.2) | 0.6406 | 27.4 | 6.2 (3.7) | 0.0946 | 31.4 | 2.6 (3.3) | 0.4457 |
| TJC | 25.5 | 1.8 (1.6) | 0.2767 | 1.4 | 0.0 (0.4) | 0.9123 | 27.4 | 1.7 (1.5) | 0.2454 | 27.1 | 2.9 (1.2) | **0.0208** |
| EGA | 14.3 | 6.6 (2.5) | **0.0129** | 1.7 | -0.2 (0.7) | 0.7922 | 16.8 | 6.3 (2.0) | **0.0025** | 16.0 | 7.4 (1.6) | **<0.0001** |
| CDAI | 15.3 | 1.7 (0.7) | **0.0163** | 1.1 | 0.0 (0.2) | 0.8428 | 17.4 | 1.7 (0.6) | **0.0050** | 16.6 | 2.1 (0.5) | **<0.0001** |
| **Month 12** |  |  |  |  |  |  |  |  |  |  |  |  |
| PGA | 4.0 | 8.3 (1.7) | **0.0001** | 0.5 | 1.1 (1.1) | 0.3386 | 4.3 | 7.9 (1.1) | **<0.0001** | 5.0 | 6.5 (1.0) | **<0.0001** |
| SJC | 23.3 | 5.0 (7.8) | 0.5263 | 3.7 | -1.9 (3.3) | 0.5811 | 17.5 | 7.5 (6.2) | 0.2367 | 16.2 | 4.6 (3.2) | 0.1632 |
| TJC | 20.4 | 4.2 (2.2) | 0.0764 | 3.4 | -0.2 (1.0) | 0.8554 | 15.1 | 4.0 (1.7) | **0.0311** | 13.9 | 3.7 (1.3) | **0.0083** |
| EGA | 13.2 | 8.8 (2.9) | **0.0074** | 2.5 | 0.5 (1.5) | 0.7581 | 9.0 | 9.1 (2.2) | **0.0003** | 9.4 | 7.4 (1.7) | **0.0002** |
| CDAI | 12.1 | 2.5 (0.8) | **0.0046** | 2.5 | 0.1 (0.4) | 0.7727 | 7.8 | 2.6 (0.5) | **<0.0001** | 8.7 | 2.0 (0.4) | **<0.0001** |
| **Month 24** |  |  |  |  |  |  |  |  |  |  |  |  |
| PGA | 12.8 | 6.0 (5.1) | 0.2628 | 12.1 | -1.7 (4.7) | 0.7165 | 1.4 | 7.9 (2.4) | **0.0059** | 2.1 | 8.2 (2.1) | **0.0008** |
| SJC | 16.2 | 33.2 (12.4) | **0.0213** | -0.6 | 41.4 (5.9) | **<0.0001** | 14.5 | -2.7 (10.2) | 0.7930 | 15.0 | -5.0 (8.7) | 0.5723 |
| TJC | 11.6 | 26.5 (5.8) | **0.0008** | -2.0 | 23.7 (4.7) | **0.0004** | 11.4 | 6.6 (6.0) | 0.2961 | 12.8 | 2.6 (4.0) | 0.5263 |
| EGA | -2.5 | 36.8 (7.8) | **0.0006** | -7.0 | 22.3 (9.6) | **0.0407** | 3.1 | 17.7 (6.6) | **0.0185** | 8.8 | 7.9 (5.6) | 0.1732 |
| CDAI | -7.9 | 9.8 (1.8) | **0.0002** | -9.5 | 5.7 (2.5) | **0.0434** | 0.4 | 4.9 (1.6) | **0.0080** | 4.8 | 3.3 (1.4) | **0.0241** |

The effect of CDAI score on WPAI score was calculated using the following linear regression model: WPAI score = Estimate of intercept + Estimate × CDAI score. Estimate indicates the incremental percent change in WPAI subscores for each single point change in CDAI score. Positive and negative estimate values correspond to an increase (i.e. worsening) or decrease (i.e. improvement) in the WPAI subscore. EGA and PGA were measured on a 10 cm visual analogue scale with 0=best and 10=worst. SJC and TJC are based on a 28-joint assessment. CDAI is a numerical sum of EGA + PGA + SJC + TJC with score ≤2.8 indicating disease remission, 2.8 to 10 indicating a low disease activity, 10 to 22 indicating a moderate disease activity, and >22 indicating a high disease activity. CDAI, Clinical Disease Activity Index; EGA, Evaluator's Global Assessment; PGA, Patient Global disease Activity estimate; PsA, psoriatic arthritis; SD, standard deviation; SJC, Swollen Joint Count; TJC, Tender Joint Count; TWPI, total work productivity impairment; WPAI, work productivity and daily activity impairment.

Supplementary Table 8. Univariate linear regression analysis of an impact of age, gender, HRQoL scores, prior biological treatment and employment at baseline on TWPI scores.

| **Variable** | **Estimate of intercept** | **Estimate (SE)** | **p-Value** |
| --- | --- | --- | --- |
| **Month 3** |  |  |  |
| Age | 11.1 | 0.4 (0.2) | 0.0794 |
| Gender (male vs female) | 26.9 | 6.5 (5.7) | 0.2567 |
| ASQoL score | 12.7 | 4.3 (0.8) | **<0.0001** |
| RAQoL score | 7.5 | 3.9 (0.5) | **<0.0001** |
| PsAQoL score | 11.6 | 3.3 (0.7) | **0.0001** |
| Prior biologics (yes vs no) | 35.0 | -5.0 (8.0) | 0.5318 |
| Employed at baseline (yes vs no) | 30.6 | 1.4 (13.2) | 0.9177 |
| **Month 12** |  |  |  |
| Age | 19.7 | 0.1 (0.3) | 0.7823 |
| Gender (male vs female) | 19.0 | 9.0 (5.9) | 0.1314 |
| ASQoL score | 14.4 | 3.3 (1.2) | **0.0126** |
| RAQoL score | 9.7 | 3.6 (0.5) | **<0.0001** |
| PsAQoL score | 6.2 | 4.6 (0.5) | **<0.0001** |
| Prior biologics (yes vs no) | 33.6 | -11.6 (9.8) | 0.2401 |
| Employed at baseline (yes vs no) | 23.9 | -11.4 (12.7) | 0.3727 |
| **Month 24** |  |  |  |
| Age | -3.5 | 0.5 (0.3) | 0.1514 |
| Gender (male vs female) | 14.5 | 6.2 (7.0) | 0.3769 |
| ASQoL score | 20.3 | 0.5 (4.0) | 0.9039 |
| RAQoL score | 8.9 | 2.2 (1.2) | 0.0927 |
| PsAQoL score | 15.7 | 2.9 (1.8) | 0.1282 |
| Prior biologics (yes vs no) | 37.5 | -24.1 (8.7) | **0.0080** |
| Employed at baseline (yes vs no) | 17.0 | 23.0 (24.2) | 0.3477 |

The effect of different variables on TWPI score was calculated using the following linear regression model: TWPI score = Estimate of intercept + Estimate × Value of variable (for continuous variables) and TWPI score = Estimate of intercept (reference value) + Estimate (for categorical variables).

Supplementary Table 9. Univariate linear regression analysis of an impact of age, gender, HRQoL scores, prior biological treatment and employment at baseline on activity impairment.

| **Variable** | **Estimate of intercept** | **Estimate (SE)** | **p-Value** |
| --- | --- | --- | --- |
| **Month** **3** |  |  |  |
| Age | 3.8 | 0.5 (0.1) | **<0.0001** |
| Gender (male vs female) | 25.7 | 6.9 (3.7) | 0.0623 |
| ASQoL score | 8.2 | 4.2 (0.4) | **<0.0001** |
| RAQoL score | 14.3 | 2.2 (0.3) | **<0.0001** |
| PsAQoL score | 13.9 | 3.1 (0.5) | **<0.0001** |
| Prior biologics (yes vs no) | 30.5 | -0.6 (4.5) | 0.8854 |
| Employed at baseline (yes vs no) | 26.9 | 8.9 (3.7) | **0.0173** |
| **Month 12** |  |  |  |
| Age | 16.8 | 0.0 (0.1) | 0.7229 |
| Gender (male vs female) | 18.9 | 0.2 (3.6) | 0.9494 |
| ASQoL score | 9.4 | 4.3 (0.6) | **<0.0001** |
| RAQoL score | 7.0 | 2.9 (0.3) | **<0.0001** |
| PsAQoL score | 8.8 | 2.8 (0.6) | **<0.0001** |
| Prior biologics (yes vs no) | 15.9 | 3.9 (4.4) | 0.3814 |
| Employed at baseline (yes vs no) | 20.0 | -2.5 (3.6) | 0.4922 |
| **Month 24** |  |  |  |
| Age | 0.1 | 0.2 (0.1) | **0.0146** |
| Gender (male vs female) | 10.8 | 1.3 (2.8) | 0.6437 |
| ASQoL score | 4.9 | 3.7 (0.7) | **<0.0001** |
| RAQoL score | 5.8 | 1.9 (0.3) | **<0.0001** |
| PsAQoL score | 6.4 | 3.4 (0.6) | **<0.0001** |
| Prior biologics (yes vs no) | 10.4 | 1.6 (3.3) | 0.6316 |
| Employed at baseline (yes vs no) | 9.8 | 4.3 (2.8) | 0.1212 |

The effect of different variables on activity impairment score was calculated using the following linear regression model: Activity impairment score = Estimate of intercept + Estimate × Value of variable (for continuous variables) and Activity impairment score = Estimate of intercept (reference value) + Estimate (for categorical variables).

Supplementary Table 10. QoL scores at each study visit. Higher score indicates worse QoL (scores range from 0 to 30 for RAQoL; from 0 to 18 for ASQoL; and from 0 to 20 for PsAQoL).

| **QoL score** | **RAQoL** | | | **ASQoL** | | | **PsAQoL** | | |
| --- | --- | --- | --- | --- | --- | --- | --- | --- | --- |
|  | **N** | **Mean (SD)** | **p-Value** | **N** | **Mean (SD)** | **p-Value** | **N** | **Mean (SD)** | **p-Value** |
| **Baseline** | 93 | 16.0 (8.0) |  | 66 | 9.2 (4.3) |  | 68 | 11.0 (5.4) |  |
| **Month 3** | 84 | 7.4 (6.4) |  | 57 | 4.1 (4.4) |  | 57 | 6.0 (5.5) |  |
| Change from baseline | 83 | -8.9 (7.4) | **<0.0001** | 55 | -5.0 (4.0) | **<0.0001** | 56 | -5.3 (5.9) | **<0.0001** |
| **Month 6** | 74 | 5.3 (5.2) |  | 45 | 3.1 (3.8) |  | 45 | 4.3 (5.2) |  |
| Change from baseline | 73 | -10.4 (7.6) | **<0.0001** | 44 | -6.0 (3.9) | **<0.0001** | 44 | -6.1 (5.7) | **<0.0001** |
| **Month 12** | 66 | 4.3 (5.1) |  | 39 | 2.4 (4.3) |  | 33 | 3.4 (4.5) |  |
| Change from baseline | 65 | -11.3 (8.4) | **<0.0001** | 39 | -6.6 (4.4) | **<0.0001** | 32 | -7.1 (6.1) | **<0.0001** |
| **Month 18** | 59 | 4.5 (5.4) |  | 27 | 2.0 (3.3) |  | 24 | 2.4 (4.3) |  |
| Change from baseline | 58 | -11.1 (7.6) | **<0.0001** | 27 | -7.3 (3.9) | **<0.0001** | 24 | -9.0 (6.4) | **<0.0001** |
| **Month 24** | 53 | 3.2 (3.8) |  | 22 | 1.2 (2.3) |  | 23 | 2.8 (5.1) |  |
| Change from baseline | 53 | -12.6 (7.5) | **<0.0001** | 22 | -8.0 (4.3) | **<0.0001** | 23 | -8.3 (6.4) | **<0.0001** |

p-values are given for comparison with baseline. QoL, quality of life; ASQoL, Ankylosing Spondylitis Quality of Life questionnaire; PsAQoL, Psoriatic Arthritis Quality of Life questionnaire; RAQoL, Rheumatoid Arthritis Quality of Life questionnaire; SD, standard deviation.
