# Supplementary material for: A Prospective Study to Evaluate the Impact of Golimumab Therapy on Work Productivity and Activity, and Quality of Life in Patients With Rheumatoid Arthritis, Psoriasis Arthritis and Axial Spondyloarthritis in a Real Life Setting in AUSTRIA. The GO-ACTIVE Study
Source: Front Med (Lausanne). 2022 Jun 2;9:881943. doi: 10.3389/fmed.2022.881943 (PMC9201205; doi:10.3389/fmed.2022.881943)
Supplement: Supplementary file 2 [file Data_Sheet_2.pdf]

# Supplementary file 2

## Work Productivity and Activity Impairment Questionnaire: Rheumatoid Arthritis

### Fragebogen zur Arbeitsproduktivität und Beeinträchtigung der Aktivität: Rheumatoide Arthritis V 2.0 (WPAI:RA)

Die folgenden Fragen beziehen sich auf die Auswirkungen Ihrer rheumatoiden Arthritis auf Ihre Fähigkeit, zu arbeiten und normalen Aktivitäten nachzugehen.

*Antworten bitte in die freien Stellen eintragen oder eine Zahl einkreisen, je nachdem.*

**1. Stehen Sie derzeit in einem Arbeitsverhältnis (bezahlte Tätigkeit)?** **NEIN** **JA**

Falls NEIN, „NEIN" ankreuzen und zu Frage 6 weitergehen.

Die nächsten Fragen beziehen sich auf **die vergangenen sieben Tage** (den heutigen Tag nicht eingeschlossen).

**2. Wie viele Stunden fehlten Sie in den vergangenen sieben Tagen bei der Arbeit aufgrund von Problemen im Zusammenhang mit Ihrer rheumatoiden Arthritis?**

*Alle Stunden einbeziehen, die Sie aufgrund Ihrer rheumatoiden Arthritis an Krankenstandstagen, Tagen, an denen Sie später kamen oder früher gingen etc., fehlten. Rechnen Sie die Zeit, die Sie wegen der Teilnahme an dieser Studie fehlten, bitte nicht ein.*

**Stunden**

**3. Wie viele Stunden fehlten Sie in den vergangenen sieben Tagen aus anderen Gründen bei der Arbeit, z. B.: Urlaub, Feiertage, freigenommene Zeit für die Teilnahme an der Studie?**

**Stunden**

**4. Wie viele Stunden haben Sie in den vergangenen sieben Tagen tatsächlich gearbeitet?**

*(Falls „0", zu Frage 6 weitergehen.)*

**Stunden**

5. **Wie sehr wirkte sich Ihre rheumatoide Arthritis in den vergangenen sieben Tagen auf Ihre Produktivität während der Arbeit aus?**

*Denken Sie hierbei an Tage, an denen das Arbeitspensum oder die Art der Arbeit, die Sie ausführen konnten, eingeschränkt war, Tage, an denen Sie weniger leisten konnten als Sie wollten, oder Tage, an denen Sie Ihre Arbeit nicht so sorgfältig wie sonst erledigen konnten. Falls sich die rheumatoide Arthritis nur wenig auf Ihre Arbeit auswirkte, wählen Sie eine niedrige Zahl. Wählen Sie eine hohe Zahl, falls sich die rheumatoide Arthritis stark auf Ihre Arbeit auswirkte.*

Bitte berücksichtigen Sie nur, wie sehr sich die rheumatoide Arthritis auf Ihre Produktivität während der Arbeit auswirkte.

| Rheumatoide Arthritis hatte keinen Einfluss auf meine Arbeit | 0 | 1 | 2 | 3 | 4 | 5 | 6 | 7 | 8 | 9 | 10 | Rheumatoide Arthritis hielt mich völlig von der Arbeit ab |
|---|---|---|---|---|---|---|---|---|---|---|---|---|

Eine Zahl einkreisen

6. **Wie sehr wirkte sich Ihre rheumatoide Arthritis in den vergangenen sieben Tagen auf Ihre normalen täglichen Aktivitäten aus (Berufstätigkeit ausgenommen)?**

*Mit normalen Aktivitäten meinen wir die üblichen Aktivitäten wie Hausarbeit, Einkaufen, Kinderversorgung, Sport, Lernen etc. Denken Sie hierbei an Zeiten, an denen das Pensum oder die Art der Aktivität, die Sie ausführen konnten, eingeschränkt war, und die Zeiten, in denen Sie weniger tun konnten als Sie wollten. Falls sich die rheumatoide Arthritis nur wenig auf Ihre Aktivitäten auswirkte, wählen Sie eine niedrige Zahl. Wählen Sie eine hohe Zahl, falls sich die rheumatoide Arthritis stark auf Ihre Aktivitäten auswirkte.*

Bitte berücksichtigen Sie nur, wie sehr sich die rheumatoide Arthritis auf Ihre normalen täglichen Aktivitäten (Berufstätigkeit ausgenommen) auswirkte.

| Rheumatoide Arthritis hatte keinen Einfluss auf meine täglichen Aktivitäten | 0 | 1 | 2 | 3 | 4 | 5 | 6 | 7 | 8 | 9 | 10 | Rheumatoide Arthritis hielt mich völlig von meinen täglichen Aktivitäten ab |
|---|---|---|---|---|---|---|---|---|---|---|---|---|

Eine Zahl einkreisen

# Work Productivity and Activity Impairment Questionnaire: Axial Spondyloarthritis

## Fragebogen zur Arbeitsproduktivität und Beeinträchtigung der Aktivität: Morbus Bechterew (MB) V2.1 (WPAI:SpA)

In den folgenden Fragen geht es um die Auswirkung Ihres MB auf Ihre Fähigkeit, zu arbeiten und Ihre Alltagsaktivitäten zu bewältigen.

*Bitte tragen Sie die geforderte Information ein oder kreisen Sie die entsprechende Zahl ein, wie angegeben.*

**1. Arbeiten Sie momentan (bezahlte Arbeit)?** **NEIN** **JA**

*Falls NEIN, kreuzen Sie bitte „NEIN“ an und fahren Sie mit Frage 6 fort.*

Die nächsten Fragen betreffen die **letzten sieben Tage,** ausgenommen heute.

**2. Wie viele Arbeitsstunden haben Sie in den letzten sieben Tagen auf Grund von Problemen versäumt, die mit Ihrem MB zusammenhängen?**

*Berücksichtigen Sie hier Stunden, die Sie auf Grund Ihres MB versäumt haben: an Krankentagen, Verspätungen, vorzeitiges Nachhausegehen usw. Zählen Sie die Stunden, die Sie wegen der Teilnahme an dieser Studie versäumt haben, nicht dazu.*

**Stunden**

**3. Wie viele Arbeitsstunden haben Sie in den letzten sieben Tagen aus anderen Gründen (wie z. B. Urlaub, Feiertage, Zeit zur Teilnahme an dieser Studie) versäumt?**

**Stunden**

**4. Wie viele Stunden haben Sie in den letzten sieben Tagen tatsächlich gearbeitet?**

*(Falls „0“, fahren Sie mit Frage 6 fort.)*

**Stunden**

**5. Wie stark hat sich Ihr MB in den letzten sieben Tagen auf Ihre Produktivität bei der Arbeit ausgewirkt?**

*Denken Sie dabei an Tage, an denen Sie in der Menge oder Art der Arbeit, die Sie schaffen konnten, eingeschränkt waren, Tage, an denen Sie weniger geschafft haben als Sie wollten, oder Tage, an denen Sie Ihre Arbeit nicht so sorgfältig wie üblich erledigen konnten. Wenn sich der MB nur geringfügig auf Ihre Arbeit ausgewirkt hat, wählen Sie eine niedrige Zahl. Wenn sich der MB stark auf Ihre Arbeit ausgewirkt hat, wählen Sie eine hohe Zahl.*

Bitte berücksichtigen Sie dabei ausschließlich, wie sehr sich Ihr MB auf die Produktivität bei der Arbeit ausgewirkt hat.

| Der MB hatte keine Auswirkung auf meine Arbeit | 0 | 1 | 2 | 3 | 4 | 5 | 6 | 7 | 8 | 9 | 10 | Der MB hat mich völlig am Arbeiten gehindert |
|---|---|---|---|---|---|---|---|---|---|---|---|---|

Eine Zahl einkreisen

**6. Wie stark hat sich Ihr MB in den letzten sieben Tagen auf Ihre Fähigkeit ausgewirkt, Ihren normalen täglichen Aktivitäten nachzugehen, ausgenommen Berufstätigkeit?**

*Unter normalen Aktivitäten verstehen wir die üblichen Aktivitäten, die Sie erledigen, wie z. B. Hausarbeit, Einkaufen, Kinderbetreuung, Gymnastik/körperliche Bewegung, Lernen usw. Denken Sie dabei an Zeiten, als Sie in der Menge oder Art der Aktivitäten, die Sie erledigen konnten, eingeschränkt waren, und Zeiten, als Sie weniger schafften als Sie wollten. Wenn sich der MB nur geringfügig auf Ihre Aktivitäten ausgewirkt hat, wählen Sie eine niedrige Zahl. Wenn sich der MB stark auf Ihre Aktivitäten ausgewirkt hat, wählen Sie eine hohe Zahl.*

Bitte berücksichtigen Sie dabei ausschließlich, wie sehr sich Ihr MB auf Ihre Fähigkeit ausgewirkt hat, Ihren normalen täglichen Aktivitäten nachzugehen, ausgenommen Berufstätigkeit.

| Der MB hatte keine Auswirkung auf meine täglichen Aktivitäten | 0 | 1 | 2 | 3 | 4 | 5 | 6 | 7 | 8 | 9 | 10 | Der MB hat mich völlig an meinen täglichen Aktivitäten gehindert |
|---|---|---|---|---|---|---|---|---|---|---|---|---|

Eine Zahl einkreisen

## Work Productivity and Activity Impairment Questionnaire: Psoriatic Arthritis

# Fragebogen zur Arbeitsproduktivität und Beeinträchtigung der Aktivität: Psoriasis-Arthritis V2.1 (WPAI:PsA)

In den folgenden Fragen geht es um die Auswirkung Ihrer Psoriasis-Arthritis auf Ihre Fähigkeit, zu arbeiten und Ihre Alltagsaktivitäten zu bewältigen.

*Bitte tragen Sie die geforderte Information ein oder kreisen Sie die entsprechende Zahl ein, wie angegeben.*

**1. Arbeiten Sie momentan (bezahlte Arbeit)?** ○ **NEIN** ○ **JA**

*Falls NEIN, kreuzen Sie bitte „NEIN" an und fahren Sie mit Frage 6 fort.*

Die nächsten Fragen betreffen die **letzten sieben Tage,** ausgenommen heute.

**2. Wie viele Arbeitsstunden haben Sie in den letzten sieben Tagen auf Grund von Problemen versäumt, <u>die mit Ihrer Psoriasis-Arthritis zusammenhängen?</u>**

*Berücksichtigen Sie hier Stunden, die Sie auf Grund Ihrer Psoriasis-Arthritis versäumt haben: an Krankentagen, Verspätungen, vorzeitiges Nachhausegehen usw. Zählen Sie die Stunden, die Sie wegen der Teilnahme an dieser Studie versäumt haben, nicht dazu.*

____ **Stunden**

**3. Wie viele Arbeitsstunden haben Sie in den letzten sieben Tagen aus anderen Gründen (wie z. B. Urlaub, Feiertage, Zeit zur Teilnahme an dieser Studie) versäumt?**

____ **Stunden**

**4. Wie viele Stunden haben Sie in den letzten sieben Tagen tatsächlich gearbeitet?**

*(Falls „0", fahren Sie mit Frage 6 fort.)*

____ **Stunden**

5. **Wie stark hat sich Ihre Psoriasis-Arthritis in den letzten sieben Tagen auf Ihre Produktivität bei der Arbeit ausgewirkt?**

*Denken Sie dabei an Tage, an denen Sie in der Menge oder Art der Arbeit, die Sie schaffen konnten, eingeschränkt waren, Tage, an denen Sie weniger geschafft haben als Sie wollten, oder Tage, an denen Sie Ihre Arbeit nicht so sorgfältig wie üblich erledigen konnten. Wenn sich die Psoriasis-Arthritis nur geringfügig auf Ihre Arbeit ausgewirkt hat, wählen Sie eine niedrige Zahl. Wenn sich die Psoriasis-Arthritis stark auf Ihre Arbeit ausgewirkt hat, wählen Sie eine hohe Zahl.*

Bitte berücksichtigen Sie dabei ausschließlich, wie sehr sich Ihre Psoriasis-Arthritis auf die Produktivität bei der Arbeit ausgewirkt hat.

| Die Psoriasis-Arthritis hatte keine Auswirkung auf meine Arbeit | 0 | 1 | 2 | 3 | 4 | 5 | 6 | 7 | 8 | 9 | 10 | Die Psoriasis-Arthritis hat mich völlig am Arbeiten gehindert |
|---|---|---|---|---|---|---|---|---|---|---|---|---|

Eine Zahl einkreisen

6. **Wie stark hat sich Ihre Psoriasis-Arthritis in den letzten sieben Tagen auf Ihre Fähigkeit ausgewirkt, Ihren normalen täglichen Aktivitäten nachzugehen, ausgenommen Berufstätigkeit?**

*Unter normalen Aktivitäten verstehen wir die üblichen Aktivitäten, die Sie erledigen, wie z. B. Hausarbeit, Einkaufen, Kinderbetreuung, Gymnastik/körperliche Bewegung, Lernen usw. Denken Sie dabei an Zeiten, als Sie in der Menge oder Art der Aktivitäten, die Sie erledigen konnten, eingeschränkt waren, und Zeiten, als Sie weniger schafften als Sie wollten. Wenn sich die Psoriasis-Arthritis nur geringfügig auf Ihre Aktivitäten ausgewirkt hat, wählen Sie eine niedrige Zahl. Wenn sich die Psoriasis-Arthritis stark auf Ihre Aktivitäten ausgewirkt hat, wählen Sie eine hohe Zahl.*

Bitte berücksichtigen Sie dabei ausschließlich, wie sehr sich Ihre Psoriasis-Arthritis auf Ihre Fähigkeit ausgewirkt hat, Ihren normalen täglichen Aktivitäten nachzugehen, ausgenommen Berufstätigkeit.

| Die Psoriasis-Arthritis hatte keine Auswirkung auf meine täglichen Aktivitäten | 0 | 1 | 2 | 3 | 4 | 5 | 6 | 7 | 8 | 9 | 10 | Die Psoriasis-Arthritis hat mich völlig an meinen täglichen Aktivitäten gehindert |
|---|---|---|---|---|---|---|---|---|---|---|---|---|

Eine Zahl einkreisen

Reilly MC, Zbrozek AS, Dukes EM. The validity and reproducibility of a work productivity and activity impairment instrument. Pharmacoeconomics. 1993 Nov;4(5):353-65.
